# A grounded theory on acceptance of diagnosis as a pathway to recovery in bipolar disorder

**DOI:** 10.1038/s41598-024-61923-5

**Published:** 2024-06-11

**Authors:** Caroline Silveira Pereira, Carolina Stopinski Padoan, Marielle Moro Silva, Pedro V. S. Magalhães

**Affiliations:** Graduate Program in Psychiatry and Behavioral Sciences, Faculty of Medicine, Hospital de Clínicas de Porto Alegre, Centro de Pesquisa Clínica, Universidade Federal do Rio Grande do Sul, Porto Alegre, Brazil

**Keywords:** Human behaviour, Quality of life

## Abstract

The recovery process in bipolar disorder is a subjective and multidimensional experience that seeks to develop new meanings and purposes for living a satisfying life despite the limitations imposed by the disorder. Thus, this qualitative study aimed to explore the perceptions of recovery and the meanings attributed by individuals undergoing treatment for bipolar disorder to the elements considered relevant in this process. Semi-structured interviews with open-ended questions were conducted to explore the experiences and perspectives of recovery in individuals undergoing treatment for bipolar disorder. Grounded Theory was used as the method for qualitative analysis. The study included 26 participants aged between 18 and 65 years. Based on the analysis of participant reports, we identified two main themes: living with the illness and what it means to be in recovery. The perception of recovery is an individual process and can differ from the medical model. Participants suggest that accepting the diagnosis of bipolar disorder and finding meaning in life are essential to their recovery. They also describe how mental health professionals can facilitate or hinder this process. Understanding patients’ perceptions can facilitate access to healthcare services and treatment adherence.

## Introduction

Bipolar disorder is a serious and debilitating condition characterized by recurrent episodes of depression and mania or hypomania. With a chronic course, it negatively impacts functioning, interpersonal relationships, and personal identity^1–9^. Bipolar disorder is a lifelong condition and the existing literature describes the difficulty in accepting the diagnosis, adhering to treatment, and coping with symptoms, as well as the impact this has on their identity, autonomy, functionality, and confidence to rethink their goals in a way that is compatible with their new reality^5,9–11^. Although the illness is chronic, individuals may perceive themselves as recovered or in recovery^12^. Recovery is a continuous and deeply personal process of regaining control of one’s identity and life, changing attitudes, goals, skills, and roles^13–15^; it is a subjective and multidimensional experience that recognizes the social and structural determinants of health and seeks to learn to live with the illness and striving for a balance between feeling recovered and at the same time, vulnerable to the illness and its limitations and to develop new meanings and purposes for living a full and meaningful life for oneself^16–18^.

According to the World Health Organization^19^, good mental health practices should be oriented toward person-centered recovery, respecting patients’ experiences and choices, and supported by an expanded care network that is attentive to social determinants. This model acknowledges the importance of listening to and considering the perceptions of those living with mental illness because as the treatment makes sense to that person, the responses tend to be more effective^13^. Additionally, it encourages self-awareness and contributes to developing autonomy and a sense of responsibility for oneself, as well as building quality relationships to make care more effective^20,21^. Therefore, focusing interventions solely on the clinical perspective of the illness tends to be less effective because it does not address the multiple dimensions of a person's development^22,23^. In this sense, the difficulty in accepting the illness, negative experiences with medications and healthcare professionals, loss of autonomy and identity, internalized stigma, and lack of social support also contribute to making recovery more challenging^18,24–28^. A person-centered recovery model that respects the singularities and experiences of those living with the condition has been proposed. This model supports acceptance and self-management of the illness as a means to promote recovery and it proposes an attention focused on the person's needs and support in times of crisis^21,28–30^. International qualitative studies suggest that interventions should find a balance between pharmacological and non-pharmacological treatment, focusing beyond the illness and its symptoms. Approaches that facilitate coping with acute phases of the illness, resumption of positive relationships and social and occupational activities, understanding, management, and living well with the disorder are essential for recovery^9,20,21,31–35^.

Failure to address these issues may lead to distancing from health services and hamper treatment adherence, common problems in bipolar disorder^2^. This qualitative study aims to investigate how individuals undergoing treatment for bipolar disorder perceive recovery and the significance they attribute to it.

## Results

We included 26 participants in the study, nineteen women and seven men (supplementary material I). Based on the analysis of participant reports, we identified two main themes: (1) living with the illness, and (2) what it means to be in recovery.

### Living with the illness

We understand from the participants’ accounts that it is crucial to know what it is like to live with bipolar since recovery is a process that begins with the history of diagnosis. According to the accounts, this period tends to be lengthy and painful. It involves many uncertainties, fears about the illness and treatment, and great difficulty in understanding and accepting this condition.

“I thought I wasn’t sick. When it’s something like the heart, you know it’s the heart. The liver, you know it’s the liver, but understanding that it’s here (points to the head) is difficult, it’s really difficult, very complicated” (E02).

Therefore, the person hopes to be supported by those close to them because it is a period that requires tolerance and resilience. With the definition of the diagnosis, a possible path is a movement towards accepting the illness and constructing a new identity, accompanied by the process of mourning for the losses and limitations resulting from this condition.

“I tried to resist because I was a much more energetic person, I lived in the manic phase and then when I started taking medication, my mood became dampened. I no longer had that side that fought, but I had a side that didn’t seem like me, and I complained to the doctor. I argued with the doctor. And she said to me—look, now you’re neutral, you’re no longer up, you’re neutral, and then I said—no, I don’t want to be like this. So, I prefer to stay as I was before. I wanted to be altered, I wanted to be impulsive, and she said no, no chance. You have to treat yourself, and in the end, I had to accept it (…) I had to accept it, right. There was no other way. There comes a point where we have to accept it” (E07).

In this scenario, different processes connect treatment and recovery. The first one involves accepting the diagnosis and is mediated by health literacy and the quality of the bond with the healthcare professional. Knowing and recognizing bipolar disorder and its demands helps the individual understand the need to take on their treatment and, therefore, experience its benefits.

“I suffered a lot for about two, three years because I didn’t accept it, didn’t understand it, didn’t have knowledge. And then after that time, when I hit rock bottom, that's when I started searching, understanding, researching, reading about what was happening to me. That’s when I finally understood” (E02).

To facilitate this process, our interviewees emphasize the importance of considering different management strategies in the treatment and viewing medication as a part of the recovery process, but not the final stage.

“This influenced me to open my eyes and see that there are other options besides medication. Taking a sunbath, at least on the wrists, walking, diet. I like music, and I like to read some things too. So, adding these things to medication has been beneficial (…) if you don’t change these things, it can worsen. And medication is not everything” (E19).

Additionally, establishing a quality bond with the healthcare professional, that is, a respectful relationship with space for the joint construction of a treatment plan that can balance the person's expectations with the disease prognosis, as well as discussing recovery issues and a life plan, has the potential to bring the subject closer to their treatment and experience the feeling of recovery. In real life, coping is supported by the perspective of an integral treatment and their support network. These strategies contribute to preserving autonomy, establishing a good relationship with treatment, and the possibility of recovery.

“With each of them (doctors), I maintained this bond, and I learned more with each one because with them, I became a person. With them, I can talk about my projects, correct my course, see if I'm okay, have that confidence that with them, I can talk, I can talk about my studies, I can talk about my medications, I can talk about my whole life” (E10).

“Usually, I write down the things that I feel physically and mentally, and generally, in depressive crises, I also do these same things, I write down these things and bring them to the appointments to discuss together about what we can think or do from there” (E16).

“That's why I say—sometimes, another resource can help you get out of that thing. The doctor wanted to give me medication, but I insisted on psychotherapy, and he got me a spot. And then with the psychotherapy doctor, we were working on it. I never refused—oh, I’m not going to take the medicine, but I wanted to at least try, and then in therapy, we were going, working, until I got out of that horrible depression that I was in, and I didn’t need to use medication. I remain with the ones I already had” (E08).

Another story involves the difficulty of understanding and accepting the diagnosis, leading the individual to a more pessimistic perspective regarding the course of the illness and the outcomes of treatment. This approach brings them closer to significant limitations, making them prisoners of the symptoms and further away from the possibility of recovery. Symptoms and stressors are avoided, rather than faced.

“I used to take that medication, I would improve and then stop taking it because, as I told you, I believed I had no illness. And of course, after some time, I would relapse again” (E08).

“I believe that being able to focus only on the treatment and not worry about going out to work or face other things and problems, I think that helped” (E17).

In addition to exacerbating isolation and loss of autonomy, this path leads to a feeling of frustration and helplessness in the face of the illness, contributing to a lack of hope in oneself and the possibility of recovery.

“When I used to work, I would fix my hair, eyebrows, do my nails, but now I do nothing, I don’t take care of myself, I don’t do anything, I don’t have any desire, really. And it’s everything, not just that. I don’t go to the gym, I don’t go for walks, all of that (…) I’m already aware that it will be like this for the rest of my life. I have no hope, no objective, I don’t think it’s going to change” (E17).

### What is it like to be recovered?

For our interviewees, being recovered means establishing a good relationship with the chronicity of the illness, with the possibility of relapse and with the treatment.

“I'm not sure, but as far as I know, it’s not something that has a cure, it’s something that you’ll learn to deal with somehow, and then I think that recovery in this case is like, it has to do with the treatment issue, but it’s like, being able to align it in a way that you can live with your disorder—because it won’t disappear” (E16).

Also, being recovered means maintaining a stable mood, being reintegrated into different social roles, and accepting that the illness is a part of your life but does not define or limit you. Furthermore, being recovered means feeling capable of regaining autonomy and control of your life and of thinking and achieving goals.

“I feel recovered. I haven’t had any visions or heard anything anymore. Of course, there are days when I’m sadder (…), but I’m managing to do my things, sleeping well” (E20).

“Before, I didn’t see any color, there was no color in my life. And now there is color. So, for me, that’s already a recovery, it’s a big victory (…). My life has meaning again. I’m becoming an active person again” (E10).

Our participants indicated factors they consider crucial for recovery, and the first step of this process is accepting the diagnosis and treatment, combined with health literacy.

“Actually, I was quite relieved to have my diagnosis more accurate because then I could have confidence in a treatment that would actually work” (E16).

A quality relationship with the healthcare professional was also described as essential for recovery because it establishes a bond and facilitates the person's involvement in their treatment. In this sense, the professional should empower and invest authority in the individual for more assertive decision-making regarding their treatment, promoting the necessary protagonism and autonomy for the construction of a recovery plan.

“The healthcare professional must be humane, they must have empathy, it’s not just about giving a prescription and that’s it. And when you find a humanized professional, you feel welcomed. As I said, things that I don’t talk about with a friend, when I go to the psychiatrist, I can express myself” (E17).

“I know that the doctor has knowledge ten thousand times greater than mine, but I’m the one taking the medication, and I think that if I’m not doing well, I have the right to say that I’m not doing well until it’s adjusted” (E26).

Receiving support from important people through respectful, sensitive, and effective care is essential in the process of accepting and recovering from the illness.

“I have already had manic episodes, had about three strong depressive episodes, and was hospitalized, had episodes of self-mutilation, and it is very sad because at that moment, we do not have a perception of what we are doing to ourselves. And one very important thing to keep in mind is that having someone by your side who gives you support makes all the difference. Now I have entered a depressive phase, and my husband helped me a lot—he went to the appointment, understood, talked to me, did not force me to do anything I didn’t want to” (E07).

Recognizing signs and situations that can trigger more acute symptoms was also described as essential for recovery. For many participants, this is a reflective and challenging process because it is often difficult to separate their personal characteristics from the symptoms.

“What is the measure of normal extraversion? There is no measure. What will happen is continuity, if this euphoria remains and starts to manifest itself in strange ways that make people uncomfortable or put me at risk, but the measure of being excited about something is very similar—a normal thing of a turn. I can be super excited about something, now if it continues like this, right…” (E28).

Finally, the ability to think about objectives and plan a life in the presence of the illness and its treatment is crucial for recovery and should therefore be included in the treatment plan. To turn these objectives into a life plan, it is important to understand the illness and its prognosis and accept that despite the changes, it is possible to build a new life trajectory:

“In the exchange of medications, I was stabilizing, improving, and then I started studying again, doing projects always with the doctor who treated me. We started rebuilding together. Everything in parts so that I wouldn’t have a setback…it was all in stages, like a child. So it was all planned, happening little by little, but I started’’ (E10).

There are also factors that hinder the recovery process. According to the reports, delayed diagnosis and consequently delayed initiation of more assertive treatment, a weak therapeutic alliance, little engagement in treatment and outsourcing of care, avoidance behaviors, socio-economic and cognitive difficulties act as important obstacles to recovery. These factors contribute to loss of autonomy, excessive side effects with medications, identity excessively infiltrated by the illness, greater cognitive decline with disease progression, delay in adjusting the treatment plan and experiencing its benefits, and a sense of irrecoverable losses throughout life.

“This delay was also due to a lot of prejudice, disbelief, and lack of importance. It was also due to a lack of interest because if they had an interest, they would have gone there and asked the doctor. Many situations of hospitalization that disrupted my treatment would not have occurred” (E10).

## Discussion

Participants describe recovery as a subjective, individual, and non-linear process, with advances and setbacks. In their perception, being recovered means feeling capable of building a new way of living and coexisting with the illness in a way that does not imply losing control of their lives. This process occurs within a social and cultural context, and therefore, different factors can determine its success.

**Figure 1:**
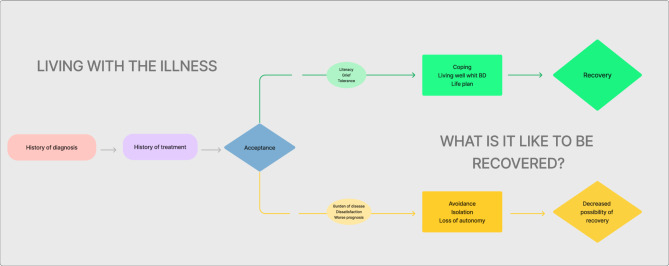
Conceptual map—the experience of recovery in bipolar disorder.

The narratives here suggest that acceptance of the diagnosis of bipolar disorder can be a relevant pathway to recovery. Those who were able to understand and accept their diagnosis were better equipped to give meaning to their lives and move toward recovery. Conversely, individuals who were more resistant to accepting their diagnosis tended to have a treatment history focused on managing symptoms and limitations, making the recovery process more challenging. The meanings attributed to recovery also emphasize the importance of stability and functioning. Accepting their condition is one path where individuals with bipolar disorder can avoid having their lives defined by the illness and instead focus on achieving stability and maintaining functioning. Furthermore, the narratives suggest that recovery also entails rebuilding one’s life and identity in the context of living with a chronic illness. Participants spoke of developing new coping strategies and re-engaging with social roles, which allowed them to move beyond their illness and redefine their sense of self. The ability to maintain autonomy and set personal goals was highlighted as key to feeling recovered. Thus, recovery from bipolar disorder can be seen as a dynamic and ongoing process that involves both managing the illness and reclaiming a fulfilling and autonomous life (Fig. 1).

To understand the recovery process, we must first understand what it is like to live with bipolar disorder. The beginning of this journey is usually marked by experiences with different diagnoses and treatments, which requires the person to have a capacity for tolerance and resilience. When a diagnosis is delayed, it can cause a cascade of negative outcomes, including uncertainty, anxiety, and doubt. That's why it's crucial for the person to have access to a supportive network during this waiting period, to offer help and guidance^9,21,30,33^. The participants indicate that the process of accepting the diagnosis is a difficult one. The fact that the illness is “not something that appears on a test” (E10) makes this condition more abstract and difficult to understand. Walsh et al.^9^ suggest that the experience of this moment can elicit a range of emotions that vary depending on an individual’s awareness and motivation. This is supported by Warwick et al.^21^ and by the accounts of our interviewees, who stress the importance of understanding what bipolar disorder is, how it manifests, and what possible implications it may have on their lives in order to create a more favorable environment for accepting this diagnosis.

Accepting the diagnosis involves questioning how to deal with this new identity, as the sense of identity is impacted by mood swings and internalization of stigma^5,27^. The frustration of feeling different and the powerlessness in the face of the illness provide a favorable environment for the identity to be infiltrated by the diagnosis, making it difficult to separate symptoms and personality traits and driving the loss of autonomy and distancing these individuals from their social role. Recognizing one's limitations and nurturing positive traits can contribute to redefining identity from the perspective of recovery^17,25,27,31^. Understanding and accepting the diagnosis and this new identity impact the understanding that it is necessary to engage in treatment that best offers means to regain quality of life and contribute to recovery. In the perception of our interviewees, as well as the findings of Maassen et al.^33^, good treatment is one that not only assists in (re)building autonomy but also ensures the individual that the adopted behaviors are beneficial. In this context, the treatment history is mediated by acceptance and the ability to tolerate unwanted effects, the mourning for euphoria, and the time required to experience its benefits.

Approaches should include different coping strategies that incorporate the perspectives of those who have the disease. For our participants, it is important to establish a respectful bond with the healthcare professional. According to the findings of Tse et al.^36^, for many people with bipolar disorder, empathy is as important as technical knowledge. Our interviewees also consider it important to jointly build a treatment plan with space to discuss issues related to the recovery process and a life project. Even though some people deal with the disease by avoiding stressors, coping with symptoms enables the individual to learn about their illness, how to take care of themselves, and develop good living with their bipolar disorder from the perspective of recovery^9,11,28,31^. As a personal process, recovery has different meanings. However, a common point put forward by our participants is about the perception that being recovered means returning to life—not necessarily to life before diagnosis but building a new way of living without feeling nullified by the disease. Our interviewees report feeling recovered by regaining their autonomy and control over their lives. In their perception, being recovered means feeling like a person again (not just a patient) with the conditions to (re)establish bonds, have a purpose in life, and celebrate their achievements. Recovery goes beyond symptom reduction and mood stability; it also means finding a way to live with the illness and its darkest moments. This perspective is consistent with current literature that discusses recovery beyond clinical improvement and sees it as a process that encourages self-acceptance, empowerment, and confidence to recalculate one’s path and regain control of their life in their different social roles^11,21,23,28,34,37^. It should be noted that symptom remission and relapse prevention are very important steps in this process, but proposing isolated pharmacological interventions can compromise the success of recovery from the subject's perspective^18,38^.

The determinants of recovery also vary according to the individual's social and cultural context^39^. Based on their experiences, our interviewees indicated themes that they perceive as crucial to their recovery: acceptance, self-knowledge about their bipolar disorder, quality of the relationship with the healthcare professional, support from important people, and the possibility of establishing a life plan. According to Russell and Browne^40^, accepting the diagnosis is the first step towards recovery. It involves letting go of what cannot be controlled to rebuild a good relationship with oneself and the external world. Mediated by literacy, it is the turning point in the recovery process^9,17,27,33^. From the perspective of recovery, it is essential for people with the illness to develop or regain their autonomy and take a leading role in their lives. Therefore, it is beneficial for the healthcare professional to be available to empower and invest authority in the subject for more assertive decision-making regarding their treatment and construction of their life plan, aligning their expectations with their prognosis.

The diagnosis and symptoms of the disease often impact the interpersonal relationships of these individuals, resulting in situations of isolation, infantilization, and significant limitations. The possibility of restoring relationships and bringing family and close people closer to this process is also seen as an important determinant for recovery. According to reports, in addition to the sense of belonging, it provides a sense of support and security to accept the illness, overcome moments of crisis, and conduct treatment toward recovery. A network capable of offering emotional support and effective assistance supports and also encourages living with the illness with greater autonomy and protagonism^9,17,21,33^. Regarding the determinants for recovery, our interviewees stated that it is important to have a life plan. That is, having the ability to think about life goals and project the future with the illness and treatment, within possible expectations. According to the findings of Lee et al.^41^, people who can find a purpose in their lives tend to be more active and engaged. The main aspirations mentioned by our interviewees were to be able to take care of themselves and their dependents, regain their ability to produce, and maintain clinical stability. In the context of recovery, participants shed light on situations that act as obstacles: delay in diagnosis and treatment, fragile therapeutic alliance, socioeconomic difficulties, lack of engagement in treatment and outsourcing of care, as well as avoidance behaviors and, in some cases, cognitive difficulties. In addition to delaying or making the recovery process unfeasible, these situations add to the overall quality of life impairments of these individuals, highlighting the need to focus attention on developing care strategies to address them. According to the reports, once these individuals found support in one of the determinants of recovery, they were able to overcome their obstacles and gradually regain their identity and rediscover their potential. Therefore, our results suggest that thinking about recovery only from a clinical perspective can compromise the success of treatment.

### Limitations

Among the main limitations of our study is the fact that all individuals were treated for bipolar disorder in a tertiary facility. Such patients usually have to surpass a series of barriers related to access to care, such as a relatively good safety net and communication skills. This may have influenced their stories of recovery, and we are certainly not claiming diagnostic acceptance is the only pathway to recovery. There are studies that report, for instance, on the experience of recovery without medication, indicating a complex role for treatment^25,42^. Participants not currently using medications commonly attribute the recovery to a “shift in perspective”, where they can find their own solutions and often redefine themselves, dispensing with diagnostic labels altogether. While we were mostly interested in experiences of recovery, we recognize there is much to be learned from experiences of non-recovery. As we excluded from our sample those without any experiences of recovery, too acutely ill to participate as well as those living with disability benefits, the study is possibly little informative on such experiences.

The accounts shared here reaffirm that the process leading to recovery is related to how people live and cope with bipolar disorder. The accounts shared here reaffirm that the process leading to recovery is related to how people live and cope with bipolar disorder. The participants’ narratives reveal that recovery is not merely the alleviation of symptoms but a multifaceted process of accepting the illness, managing its chronic nature, and reclaiming a sense of agency and identity. A pivotal element is the initial acceptance of the diagnosis, allowing individuals to engage more fully with their treatment plans and healthcare providers. A strong, respectful partnership with healthcare providers can then empower patients, fostering a sense of collaboration and trust that is essential for effective management and personal growth. Participants further articulated that incorporating activities such as physical exercise, dietary changes, and hobbies provided them with additional tools to manage their condition, thereby enhancing their quality of life and stabilizing mood fluctuations. Recovery from bipolar disorder involves a comprehensive strategy that addresses the physical, emotional, and social aspects of health.

## Method

### Design

This study aimed, through the exploration of experiences and perspectives of recovery of individuals undergoing treatment for bipolar disorder, to develop a theory to explain the processes and outcomes of such changes. We used a grounded theory approach as our methodology, which allowed us to generate a theory from the data that emerged from our participants’ experiences and perspectives^43,44^. These theories emerge inductively, that is, knowledge is generated based on the narratives of participants through the process of data collection and coding^45,46^.

Analysis occurred simultaneously with data collection and early identification of key themes in brainstorming sections allowed for refinement in the interviewing approach.

### Instrument

We employed a semi-structured interview with open-ended questions to collect data. The instrument was chosen according to the study objectives: to approach the interviewees' experiences and perceptions through open and spontaneous discourse^45,47^. A guide was constructed beforehand, indicating the main points to be explored: living with symptoms, recovery, treatment, work-related issues, lifestyle and health, social issues, and living with bipolar disorder. The guide was pilot-tested and the interview allowed the participants to tell their stories in their own words and with spontaneous associations. The interviews were conducted by a physical education professional (CP) with clinical experience and a master's degree in mental health, with previous experience conducting qualitative interviews with this population^48^. By adopting a welcoming and respectful attitude, it was possible to create a safe and conducive environment for authentic narratives. The interviews were conducted online and in person at the Clinical Research Center of the Hospital de Clínicas de Porto Alegre and lasted about 1 and a half hours. All interviews were recorded and later transcribed by the researcher.

### Participants

Forty-one people with a diagnosis of bipolar disorder aged between 18 and 65 years were invited to participate in person or through telephone contact. Of those, five refused to participate, eight failed to attend the intake interview and a further two were removed from analyses because they fulfilled exclusion criteria. The Bipolar Disorders Program at the Hospital de Clínicas de Porto Alegre is an outpatient clinic that specializes in bipolar disorder, with a tradition in clinical research. Patients are diagnosed based on the best clinical information available, and the diagnosis of bipolar disorder is a precondition to receiving care at the facility. This outpatient clinic is part of a public tertiary care hospital and offers specialized outpatient care. Our sample consisted of men and women diagnosed with bipolar disorder, mostly single and residents of the state capital or metropolitan region. Despite their level of education, many were unemployed or relied on sickness benefits provided by the National Institute of Social Security of the Federal Government as their source of income. Individuals in a severe mood episode, presenting psychotic symptoms, intellectual disability, or those who were retired due to disability were excluded from the study.

Sampling was carried out in stages^45^. In the first stage, a purposeful convenience strategy was used to collect the maximum amount of data from different individuals who had rich experiences and narratives about the subject of recovery. In the second stage, a theoretical sampling approach was used to seek narratives that could expand and develop our categories, as well as negative cases. Saturation of data was achieved through a careful and iterative process of data collection and analysis. We followed the principles of theoretical sampling^45,49^ to ensure that we collected data from a diverse range of participants who had rich experiences and narratives related to the subject of recovery from bipolar disorder. In the first stage, a purposeful convenience strategy was employed to gather a wide range of perspectives, of individuals with varying ages, gender identities, socioeconomic backgrounds, and treatment experiences. In the second stage, a theoretical sampling approach was used to seek out narratives that could further expand and develop our emerging categories, as well as identify any potential negative cases. Theoretical sampling involved deliberately selecting participants who could provide unique insights or challenge the emerging theories. The two stories on recovery presented below were uncovered and refined during this stage.

Throughout the process of data collection and analysis, we maintained detailed memos and conducted regular team meetings to discuss emerging patterns and concepts. These meetings facilitated a thorough examination of the data, and any disagreements or discrepancies were resolved through discussion and consensus among the researchers involved in the analysis.

### Data analysis

The data analysis process followed Strauss and Corbin's Grounded Theory^46^. Two researchers with extensive experience in grounded theory (CSP and CSP) conducted the analysis, ensuring a comprehensive and nuanced examination of the data. The analysis process consisted of three distinct stages: open coding, axial coding, and selective coding, as outlined by Fassinger^45^. These stages allowed for a thorough exploration of the data and the identification of key themes and categories. During the open coding stage, the researchers independently analyzed the interview transcripts, line by line, and assigned initial codes to capture the participants’ experiences and perspectives. Following the open coding stage, the researchers moved on to axial coding, which involved organizing the codes into broader categories and exploring the relationships between them. The researchers examined how the codes were related and clustered them into overarching themes and subthemes. Finally, the selective coding stage focused on selecting the core categories and concepts that best represented the central themes and theoretical insights emerging from the data. The researchers engaged in a process of constant comparison, comparing codes, categories, and concepts to refine the theory. This stage involved identifying the most significant and relevant categories, their properties, and the relationships between them. The researchers ensured that the theory captured the complexity and nuances of the participants' experiences of recovery from bipolar disorder. To support the analysis process, NVivo software^50^ was utilized as a data management tool.

### Ethical issues

All participants selected for this study were informed about the objectives, and procedures, as well as risks and benefits of participating in this research. Participants were already under specialized medical care; in case of significant risk becoming apparent during the interview, the care team was notified. The Informed Consent Form was presented to the participant for their agreement and, at the end of the interview, all participants were asked about the maintenance of their consent and invited to provide feedback on the research process. This project was submitted and approved by the Research Ethics Committee of the Hospital de Clínicas de Porto Alegre under registration number 2019-0516. We report the findings according to the current Consolidated Criteria for Reporting Qualitative Research Guidelines^51^ (supplementary material II).

### Supplementary Information

Supplementary Table 1.

Supplementary Information 2.

## Data Availability

The datasets generated during and/or analyzed during the current study are available from the corresponding author on reasonable request.
