# Supplementary material for: A grounded theory on acceptance of diagnosis as a pathway to recovery in bipolar disorder
Source: Sci Rep. 2024 Jun 11;14:13434. doi: 10.1038/s41598-024-61923-5 (PMC11166999; doi:10.1038/s41598-024-61923-5)
Supplement: Supplementary file 1 — Supplementary Table 1. [file 41598_2024_61923_MOESM1_ESM.docx]

| Participant | Gender | Age |  | Family Income | Education | | Occupation | Marital Status | | |
| --- | --- | --- | --- | --- | --- | --- | --- | --- | --- | --- |
| E01 | F | 46 |  | 1.192,40 | High School | | On disability | | Single | |
| E02 | F | 45 |  | 3.000,00 | Incomplete High School | | Housewife | | Married | |
| E03 | M | 45 |  | 2.400,00 | High school | | Unemployed | | Single | |
| E04 | M | 24 |  | 600,00 | High school | | Employed | | Single | |
| E05 | M | 48 |  | 1.914,00 | Incomplete Primary Education | | On disability | | Separated | |
| E06 | F | 45 |  | 1.192,40 | College | | On disability | | Married | |
| E07 | F | 50 |  | 1.000,00 | High School | | Unemployed | | Married | |
| E08 | F | 57 |  | 2.700,00 | High School | | Unemployed | | Married | |
| E09 | M | 27 |  | 2.400,00 | High School | | Employed | | Single | |
| E10 | F | 58 |  | 2.100,00 | College | | Retired | | Single | |
| E11 | F | 56 |  | 1.200,00 | Incomplete Primary Education | | Pensioner | | Widowed | |
| E12 | M | 36 |  | 3.500,00 | Incomplete Primary Education | | On disability | | Single | |
| E14 | F | Not mentioned |  | 400,00 | Incomplete Primary Education | | Housewife | | Separated | |
| E15 | F | 65 |  | 1.200,00 | | High School | On disability | | | Widowed |
| E16 | F | 21 |  | 1.000,00 | | Incomplete Higher Education | Student | | | Single |
| E17 | F | 50 |  | 1.900,00 | | College | Housewife | | | Separated |
| E18 | F | 37 |  | 4.000,00 | | College | Employed | | | Married |
| E19 | F | 30 |  | 3.000,00 | | College | Unemployed | | | Single |
| E20 | F | 37 |  | 1.700,00 | | High School | Employed | | | Single |
| E21 | M | 47 |  | 1.200,00 | | High School | Unemployed | | | Separated |
| E22 | F | 44 |  | 18.000,00 | | Incomplete Higher Education | Housewife | | | Single |
| E23 | F | 30 | - | 1.200,00 | | Primary Education | On disability | | | Married |
| E25 | F | 41 |  | 6.500,00 | | College | Employed | | | Single |
| E26 | M | 39 |  | 8.000,00 | | Incomplete Higher Education | Businessperson | | | Single |
| E27 | F | 35 |  | 3.900,00 | | High School | Employed | | | Married |
| E28 | F | 52 |  | Not mentioned | | College | Employed | | | Single |

**Supplementary table 1.** Characteristics of the study sample
